# Microvessel density in the placental bed among preeclampsia patients

**DOI:** 10.1590/S1516-31802006000200009

**Published:** 2006-03-02

**Authors:** Tarcisio Mota Coelho, Nelson Sass, Luiz Camano, Antonio Fernandes Moron, Rosiane Mattar, João Noberto Stávale, Maria Regina Régis Silva, Marília da Glória Martins, João Nogueira

**Keywords:** Pre-eclampsia, Proteinuria, Trophoblasts, Endothelium, Endothelins, Pré-eclâmpsia, Proteinúria, Trofoblastos, Endotélio, Endotelinas

## Abstract

**CONTEXT AND OBJECTIVE:**

Morphological changes in the spiral arteries of the placental bed have been studied in patients with preeclampsia, one of the largest causes of maternal and perinatal morbidity and mortality. The reports show that vasospasm and vascular endothelial injury were two major pathological conditions for preeclampsia. The aim of this study was to investigate the microvessel density of spiral arteries in the placental bed, in pregnancies complicated by hypertension and proteinuria, and in normal pregnancies.

**DESIGN AND SETTING:**

This was a cross-sectional survey of immunohistochemical studies on biopsies from the spiral arteries of the placental bed, among women undergoing cesarean sections for clinical and obstetrical reasons at Universidade Federal de São Paulo, São Paulo, Brazil.

**METHODS:**

Placental bed biopsies were obtained during cesarean section after placenta removal, with direct viewing of the central area of placenta insertion. The microvessel density of spiral arteries was measured by immunohistochemical methods in decidual and myometrial segments, using CD34 antibody.

**RESULTS:**

Biopsies containing spiral arteries were obtained from 34 hypertensive pregnant women with proteinuria, and 26 normotensive pregnant women. The microvessel densities in decidual and myometrial segments of the placental bed were compared between the groups. It was observed that, with increasing blood pressure and proteinuria, the microvessel density gradually decreased.

**CONCLUSION:**

The presence of high levels of hypertension and proteinuria may be associated with a progressive decrease in microvessel density in the placental bed.

## RESUMO

**CONTEXTO E OBJETIVO:**

As mudanças morfológicas nas artérias espiraladas no leito placentário têm sido exaustivamente estudadas na fisiopatologia da pré-eclâmpsia, moléstia esta que está entre as principais causas de morbidade e mortalidade materna e perinatal. Segundo estudos, o vasoespasmo generalizado e os danos endoteliais representam as principais alterações fisiopatológicas da pré-eclâmpsia. O objetivo do estudo foi avaliar a microdensidade vascular no leito placentário nas gestantes portadoras de pré-eclâmpsia comparado às gestantes normotensas e possíveis repercussões maternas e perinatais.

**TIPO DE ESTUDO E LOCAL:**

Estudo transversal em gestantes portadoras de pré-eclâmpsia e gestantes normo- tensas submetidas à operação cesariana por indicações clínicas e obstétricas realizado no Departamento de Obstetrícia da Escola Paulista de Medicina da Universidade Federal de São Paulo, no período de 1^o^ de novembro de 2003 a 30 de novembro de 2004.

**MÉTODO:**

34 gestantes portadoras de pré-eclâmpsia e 26 gestantes normotensas foram submetidas à biópsia na área central do leito placentário para exame imunoistoquímico, usando o antígeno CD 34 para avaliar a microdensidade vascular em ambos os grupos, comparando-os.

**RESULTADOS:**

Biópsia do leito placentário contendo artérias espiraladas de 34 gestantes com hipertensão e proteinúria e 26 gestantes normotensas. Foi realizada a contagem das artérias nos segmentos decidual e miometrial. A microdensidade vascular foi menor nas gestantes portadoras de pré-eclâmpsia quando comparadas às grávidas normotensas e a microdensidade foi diminuindo progressivamente à medida que os níveis pressóricos foram mais elevados bem como os níveis de proteinúria.

**CONCLUSÃO:**

As gestantes portadoras de hipertensão e proteinúria têm microdensidade vascular diminuída no leito placentário, agravada pelos níveis pressóricos e proteinúria, provavelmente diminuindo o aporte de oxigênios e nutrientes ao concepto.

## INTRODUCTION

In normal pregnancies, trophoblast cells derived from the basal plate and the tips of the anchoring villi infiltrate into the stroma of the decidual and inner myometrium. They migrate into arterial lumina and alter the internal elastic and muscle-elastic medial laminae of these arteries, in a process termed "physiological change."^1^ The placenta becomes more dilated by extensive invasion of the decidua by cytotrophoblast cells, but this is physically limited and restricted to the first 18 weeks of pregnancy.^2^

During this process, the trophoblasts come into contact with the spiral arteries, flood into the lumen and replace the endothelial cells. These physiological changes lead to reduced vascular resistance and a considerable increase in blood supply to the fetus. This trophoblast invasion appears to occur in two phases up to about the 18^th^ week of pregnancy.^2^ In preeclampsia and in some cases of intrauterine growth retardation, the physiological changes in the spiral arteries are confi ned to their decidual segments. Between one third and half of the length of the spiral arteries in the placental bed is not affected by the endovascular trophoblast invasion,^3^ and thus the myometrial segments remain intact. The reason for the incomplete trophoblast invasion of the myometrial segments of the spiral arteries that is associated with preeclampsia is unclear. The lack of physiological change in the myometrial segments of the spiral arteries in preeclampsia cases has been postulated as a factor in the development of this disease.^4^

The generalized vasospasms observed in preeclampsia case, which have major repercussions on the brain, liver and fetal-placentation unit in particular, may be an immune response to the abovementioned modifications.^5^ It has been questioned whether the microvessel density becomes altered in hypertensive pregnant women with proteinuria, and with respect to diastolic blood pressure and proteinuria levels.^6^

## OBJECTIVE

To compare the microvessel densities in the central placental insertion area known as the placental bed, in pregnancies with hypertension, according to the diastolic blood pressure and proteinuria levels, and in normal pregnancies.

## MATERIALS AND METHODS

This was a cross-sectional study, performed at the maternity hospital of Universidade Federal de São Paulo, from November 1, 2003, to November 30, 2004. Women who had attended antenatal and intrapartum care and who had delivered by cesarean section were recruited after institutional ethical permission had been granted. The patients' informed consent for placental bed biopsies was obtained. Only singleton pregnancies were included for this study.

Two groups were studied: (a) pregnant women with hypertensive disorders and proteinuria (patients with underlying disorders were excluded); (b) women with normotensive pregnancies, without proteinuria or fetal growth retardation complications, who delivered between 37 and 42 weeks. The definition of preeclampsia followed the criteria of the National High Blood Pressure Education Program (NHBPEP).^7^ Proteinuria was considered to be abnormal when it was greater than 0.3 g per 24 hours.

For both groups, the clinical data assessed for the mothers were: maternal age, parity, skin color, medial blood pressure, diastolic blood pressure and proteinuria level per 24 hours. The perinatal data considered were: gestational age, birth weight and adequacy of weight for gestational age. The latter was assessed using the Alexander chart,^6^ on which the infant's birth weight was considered to be adequate for the gestational age between the 10^th^ and 90^th^ percentiles and low for gestational age when the weight was less than the 10^th^ percentile.

**Figure 1:**
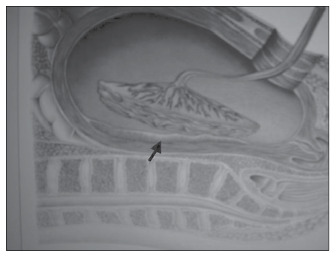
Diagram showing location for obtaining placental bed biopsy.

**Figure 2:**
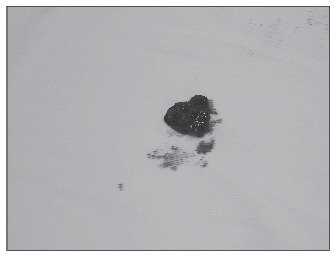
Placental bed biopsy specimen, measuring 2.5 cm across x 2.0 cm deep.

The patients were classified according to diastolic blood pressure: normotensive, with diastolic blood pressure between 80 and 90 mmHg; between 90 and 100 mmHg; between 100 and 110 mmHg; and 1 10 mmHg and above. They were further classified according to proteinuria levels: absence of proteinuria; proteinuria between 0.3 and 1.0 g; proteinuria between 1.0 and 2.0 g; and proteinuria 2.0 g or more. The placental bed biopsy was performed during the cesarean section, by using curved scissors in the central area, under direct vision after removal of the placenta (Figure 1). The wedge-shaped biopsies thus obtained measured approximately 2.5 cm across x 2.0 cm deep (Figure 2). The site was inspected for bleeding and a suture was inserted to secure homeostasis, if necessary.

**Figure 3:**
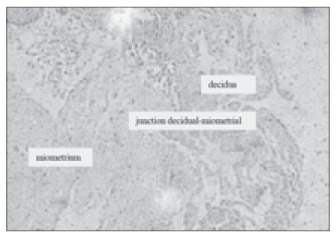
Junction between the decidua and myometrium in a placental specimen (CD34; 200 x).

**Figure 4:**
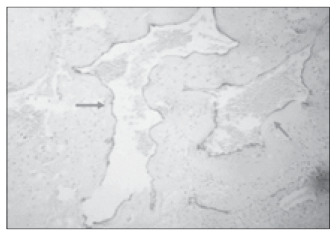
Spiral arteries in the placental bed at term; extremely dilated lumen (CD34; 400 x).

The specimens were immersed in 10% buffered formaldehyde. Then, sections of 3 µm in thickness were cut and made into slides. These were processed for hematoxylin-eosin staining, carried out according to conventional procedures. Through this, it was histologically confirmed that these were true placental bed biopsies, as identified by the presence of decidua, myometrium, extra villous trophoblast cells and the presence of spiral arteries (Figures 3 and 4).

Immunostaining was carried out using the avidin-biotin-peroxidase complex method. After deparaffinization and rehydration, endogenous peroxidase activity was blocked using 3% hydrogen peroxide in pure methanol for 10 minutes at room temperature. The tissues were then treated with 0.01% pepsin in 0.01M HCl at 37°C for 10 minutes. After serum blocking, using 2% bovine serum albumin, the sections were then incubated with the primary antibody for 30 minutes at room temperature. The primary antibody was a mouse monoclonal antibody for low molecular weight cytokeratin (clone MNF, Dako A/S, Denmark), at a dilution of 1 in 50. This was then incubated using the secondary antibody CD34 for 30 minutes.

**Figure 5:**
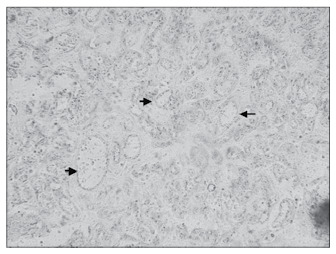
Microvessel density in the decidual portion of a placental specimen at term in normal pregnancy (CD34; 200 x).

**Figure 6:**
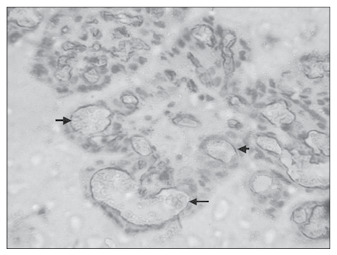
Microvessel density in the decidual portion of a placental specimen at term in normal pregnancy (CD34; 400 x).

**Figure 7:**
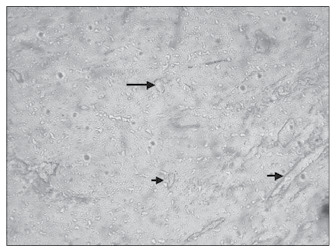
Poor microvessel density in the myometrial portion of a placental specimen at pre-term in preeclampsia case (CD34; 200 x).

**Figure 8:**
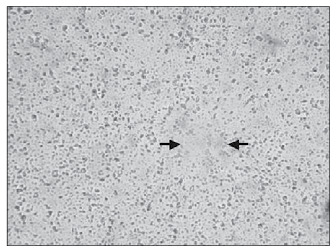
Poor microvessel density in the decidual portion of a placental specimen at pre-term in preeclampsia case, showing one vessel (CD34; 200 x).

Tissue sections were examined using an optical microscope at 200 x magnification for the initial screening. All measurements were performed at 400 x magnification. The areas of interest were recorded using a 3CCD Sony color video camera interfaced with a Nikon Optiphoto microscope. For immunohistochemical analysis, the true placental bed biopsies that showed the presence of trophoblast cells and endothelial vessels on decidual and myometrial segments were stored. Quantification was performed for the hypertensive and normotensive groups. For each specimen, ten areas of the decidua and myometrium were randomly selected and stored (Figures 5-8). The specimens were examined by the first author (T.M.C.) and supervised by two others (J.N.S. and M.R.R.S.) in two steps. The final classifications were recorded after discussion and full agreement.

Descriptive statistics on the microvessel densities in the decidual and myometrial segments were calculated and presented as mean ± standard deviation (SD). The Mann-Whitney U-test was used to make comparisons. Values were considered statistically significant at p-values < 0.05.

## RESULTS

Biopsies were obtained from 34 hypertensive patients with proteinuria and 26 normotensive patients (overall total of 60 biopsies). The microvessel densities of the spiral arteries were measured in the decidual and the myo- metrial segments of the placental bed.

The clinical classifications, demographic characteristics, clinical intercurrences and perinatal outcomes relating to the patient groups are listed in Table 1.

**Table 1:** Clinical and demographic data at delivery and perinatal outcome, among pregnant women with hypertension and proteinuria and normal pregnant women

Parameter	Hypertensive pregnant women with proteinuria n = 34	Normotensive pregnant women n = 26	p
Mean maternal age in years (± sd)	27 ± 7	29 ± 5	ns
Parity			
Nulliparous	20	6	p < 0.001
Multiparous	14	20	p < 0.001
White skin color	07	15	ns
Non-white skin color	27	11	p < 0.001
Mean diastolic blood pressure (+ sd)	124 (± 9.8)	93 (± 1.3)	p < 0.001
Mean blood pressure (± sd)	106 (± 9.4)	80 (± 1.9)	p < 0.001
Weeks of gestation (range)	32 (27-37)	38 (39-41)	p < 0.001
Mean 24-hour proteinuria (± sd)	1.6 (± 1.5)	–	
Mean birth weight (+ sd)	1755 (± 831)	3155 (± 432)	p < 0.001
AGA	18	26	p < 0.001
SGA	16	0	p < 0.001

*sd = standard deviation; ns = not significant; n = number; AGA: adequate for gestational age, defined as between the 10^th^ and 90^th^ percentile weights for gestational age in the Alexander charts; SGA: small for gestational age, defined as below the 10^th^ percentile weight for gestational age in the Alexander charts.*

It was observed that, in comparison with the normotensive patients, the group of patients with hypertension and proteinuria presented non-significantly different mean maternal age (27 ± 7 versus 29 ± 5), a greater number of primiparae (20 versus 6), a lower number of multiparae (14 versus 20) and a greater number of non-white women (27 versus 11). The mean diastolic blood pressure was 106 mmHg for the hypertensive, versus 80 mmHg for the normotensive women.

Among the patients with hypertension the mean proteinuria level was 1.6 g ± 1.5 g at 24 hours.

With regard to perinatal outcome, all the infants were born alive. Prematurity was observed in the hypertensive group with labor presented at around 32 weeks of gestation, whereas for normal pregnancies it was at about 38 weeks. In relation to birth weight, the mean fetal weight was 1,755 g in the hypertensive group and 3,155 g for normal gestation. Furthermore, the number of infants that were small for the gestational age was significantly greater in the hypertensive group (16 infants) than in the normotensive group (no infants).

The characteristics of the microvessel densities of spiral arteries in the decidual and miometrial segments of the placental bed are shown in Table 2. It can be seen that the patients with hypertension and proteinuria had lower microvessel densities than did the patients with normal blood pressure, in the decidual portion (36.5 versus 48.6) and myometrial segment (25.4 versus 38.7) The microvessel density of spiral arteries was lower in hypertensive pregnancies. With increasing diastolic blood pressures, the microvessel densities gradually decreased in both segments (Table 3). Likewise, decreased microvessel densities were observed with raised proteinuria levels, for both segments (Table 4).

**Table 2:** Microvessel density of spiral arteries in the decidual and myometrial segments of the placental bed, among pregnant women with hypertension and proteinuria and normal pregnant women

Microvessel density	Pregnant women with hypertension and proteinuria n = 34	Normal pregnant women n = 26	p
Decidual segment	36.5 ± 12.3	48.6 ± 11.8	p < 0.001
Myometrial segment	25.4 ± 11.6	38.7 ± 11.8	p < 0.001

**Table 3:** Microvessel density of spiral arteries in the decidual and myometrial segments of the placental bed, among pregnant women with hypertension according to diastolic blood pressure levels

Diastolic blood pressure	n	Decidual microvessel density	Myometrial microvessel density	p
		Mean + SD	Mean + SD	
80 90 mmHg	27	48.1 ± 13.0	38.1 ± 11.9	p < 0.001
90 100 mmHg	19	41.3 ± 14.8	27.2 ±14.3	p < 0.001
100 110 mmHg	06	33.0 ± 6.8	25.4 ± 8.3	p < 0.001
110 mmHg or more	08	27.8 ± 4.9	21.2 ± 5.3	p < 0.001

*SD = standard deviation.*

**Table 4:** Microvessel density of spiral arteries in the decidual and myometrial segments of the placental bed, among pregnant women with hypertension according to proteinuria levels

24-hour proteinuria	n	Decidual microvessel density	Myometrial Microvessel density	p
		Mean ± SD	Mean ± SD	
Absent	26	48.6 ± 13.3	38.7 ± 1.8	p < 0.001
0.3 to 1.0 g	17	40.4 ± 15.3	27.2 ± 14.4	p < 0.001
1.0 to 2.0 g	07	33.7 ± 9.2	20.9 ± 7.2	p < 0.001
2.0 g or more	10	31.4 ± 6.4	20.2 ± 4.7	p < 0.001

## DISCUSSION

The only well-documented pathophysiological event in preeclampsia is the restricted trophoblast invasion of the spiral arteries in the inner third of the myometrium. As a consequence, the physiological dilatation of the uteroplacental arteries and perfusion of the intervillous space are altered.^8,9^

The initial changes to the uteroplacental arteries involve generalized alterations of these arteries: endothelial basophilia, vacuolation, disorganized vascular smooth muscle and lumen dilatation. Structural criteria and immunohistochemical data have revealed that endovascular trophoblasts represent an end stage in the differentiation of interstitial trophoblasts derived from cell columns.^10^ It is still unclear whether or not the intravessel cells migrate into the arterial lumina.

In the present study, analysis of the demographic data and clinical characteristics of the groups showed that the maternal age of patients with preeclampsia was slightly lower than for those with normal pregnancies, with a greater number of primiparae. With respect to skin color, non-white women were a majority in the hypertensive group, and this "non-white" subgroup consisted of blacks, mulattos and those of indeterminate skin color: these data are in agreement with the literature.^11^

The microvessel density of spiral arteries in the placental bed among the hypertensive pregnant women was observed to be lower than for the normotensive pregnant women in the decidual segments (36.5 versus 48.6) and myometrial segments (25.4 versus 38.7). In addition to the presence of vascular lesions such as atherosis and medial disorganized muscular layers in such arteries,^12,13^ there was a significant decrease in vascularization in the group of patients with hypertension.

With regard to pressure levels, both the mean arterial pressure and the diastolic blood pressure were higher in the hypertensive group than in the control group (124 versus 93 and 106 versus 80, respectively). For both the decidual and myometrial segments, the microvessel density decreased with increasing pressure levels. This shows the influence of disease severity, with decreasing vascularization of the placental bed, which therefore leads to lower nutritional status for the fetus, thus contributing towards a greater numbers of infants that are small for the gestational age (SGA).

Several studies have correlated severity of the mother's clinical condition, and poor maternal and perinatal prognosis, with the proteinuria levels.^14,15^ Glomerular lesions are associated with increased blood pressure levels, probably due to endothelial activity, a physiopathological basis that leads to generalized endothelial lesions. In the study group, a mean proteinuria level of 1.6 g was observed within 24 hours. Attention is drawn to the fact that, with the presence of proteinuria, there was a significant reduction in microvessel density of spiral arteries in the placental bed, and the greater the proteinuria level was, the lower the density was.

The gestational age was lower in the group with preeclampsia (32 versus 38 weeks), the fetal weight was lower (1,755 g versus 3,155 g) and the number of SGA was greater (16 versus 0), as shown in Table 1.

Nevertheless, as the clinical severity of preeclampsia increased, in association with increased levels of hypertension and proteinuria, there was a progressive decrease in microvessel density in the placental bed. Several studies have highlighted the importance of vascular endothelial growth factor (VGEF)^16,17^ for maternal vascular irrigation, in relation to the evolution of the pregnancy. Further studies should be undertaken with the aim of analyzing microvessel density in preeclampsia cases, thereby contributing towards elucidating its etiology.

## CONCLUSIONS

The microvessel densities were very poor in hypertensive pregnancies and worsened with increasing levels of hypertension and proteinuria.
